# Diagnostic and Classification Considerations Regarding Gaming Disorder: Neurocognitive and Neurobiological Features

**DOI:** 10.3389/fpsyt.2019.00405

**Published:** 2019-06-14

**Authors:** Anthony G. Vaccaro, Marc N. Potenza

**Affiliations:** ^1^Child Study Center, Yale University School of Medicine, New Haven, CT, United States; ^2^Brain and Creativity Institute, Dornsife College of Arts and Sciences, University of Southern California, Los Angeles, CA, United States; ^3^Department of Psychiatry, Yale University School of Medicine, New Haven, CT, United States; ^4^Department of Neuroscience, Yale University School of Medicine, New Haven, CT, United States; ^5^Connecticut Council on Problem Gambling, Wethersfield, CT, United States; ^6^Connecticut Mental Health Center, New Haven, CT, United States

**Keywords:** gaming disorder, internet gaming, recreational gaming, behavioral addiction, DSM-5, ICD-11

## Abstract

Video gaming and Internet use have become a part of the everyday lives of many individuals, especially during adolescence. Given the health concerns related to problematic gaming behaviors, gaming disorder (GD) has been included in the version of the 11th edition of *The International Classification of Diseases* (ICD-11) ratified by the secretariat of the World Health Organization. Given these considerations and others (including debate regarding the most appropriate classification of GD and how best to prevent and treat the condition), there is a need for further research into GD. Specifically, we suggest that researching intermediate phenotypes focusing on cognitive and neurobiological function may help clarify GD’s relationships to other addictive disorders and more accurately define their relationships with core and associated features of GD. Overlaps in neural activity, cognitive functioning, and other features suggest that GD shares similarities with gambling and substance-use disorders and may best be classified as an addictive disorder. Individuals with GD differ from those with regular game use (RGU) on neurocognitive levels. However, concerns have been raised with respect to the differences between GD and substance-use disorders in certain dimensional features, such as tolerance. Additionally, it has been argued that differences between GD and RGU may not be fully captured by nomenclature systems like the ICD-11. Nonetheless, individuals seek treatment for help with GD, despite the limited data available for effective treatments. As more data are gathered from investigations of GD, they should be translated into refining criteria for GD and optimizing interventions.

## How Best to Define Gaming Disorder, Estimate Its Prevalence and Consider Relationships With Intermediate Phenotypes?

As the world has become increasingly “digitalized,” the prevalence of video gaming has increased substantially. As of 2016, the video game market was a 99.6 billion dollar industry and was estimated to reach 118 billion by 2019 (1). As of 2012, an estimated 1 billion people played computer games, and based on economic trends, it is likely that this number has since risen (2). Gaming is especially prevalent among children and adolescents, with an estimated 68% of 8- to 18-year-olds in the United States playing at least weekly (3). Like some other facets of technologies and their usage, gaming has, at times, come under scrutiny because of proposed links to increased violent behaviors in children, possible negative impacts on intellectual development, and lack of constructiveness. Links to aggression have been reported by some investigators to be absent or less strong than some have proposed (4), and while gaming has been reported to be linked to increased cognitive abilities in visuospatial and attention domains in some studies (5), a recent meta-analysis questions these findings (6). While most individuals game without significant concerns, there is growing evidence that some individuals may develop problematic gaming behaviors, possibly of an addictive nature. In this article, we will consider how problematic gaming has been defined in major nomenclatures systems, how different definitions have led to variations in prevalence estimates, and how investigating neurocognitive factors as potential intermediate phenotypes may help promote a better understanding of the clinical neurobiology of problematic gaming or gaming disorder (GD).

The prevalence of “addictive gaming behaviors” may vary across cultures, with estimates as low as 1.16% of adolescents in Germany, to as high as 5.9% in South Korea (7, 8), with wider prevalence estimates also noted in earlier studies (9). Estimates have varied greatly, depending on thresholds for “cases,” with estimates in adolescents, for example, ranging from 0.3% in Germany to 50% in South Korea (2). Furthermore, some studies have grouped different forms of addictive patterns of Internet use together, leading to larger estimates, such as 2.1% in Germany and 12.4% in South Korea (10, 11). As such, evaluating the prevalence of gaming problems while considering potential cultural/jurisdictional differences as well as potential differences relating to the instruments assessing gaming problems is important (12, 13).

The large range of estimates on the prevalence of problematic gaming in part relates to varying definitions. Across studies, names include “Gaming Disorder” (GD), “Gaming Addiction,” “Internet Gaming Addiction,” and “Internet Gaming Disorder” (IGD). Although the names may vary, gaming is a core behavior, and problems are a core feature. Furthermore, the terms “Internet Addiction Disorder” and related constructs may also include GD. For instance, while South Korea has officially used the term Internet addiction disorder (IAD), online gaming comprises 67% of middle school boys’ recreational use of the Internet, the group with the highest prevalence of IAD (11). The Diagnostic and Statistical Manual of Mental Disorders, 5th edition (DSM-5), published in 2013 and researched and worked on for approximately a decade prior, suggests that evidence for IGD, in part derived from existing data at the time on IAD in young males from Asian countries, may not generalize to non-gaming Internet use (14). Across researchers, the views on this potential disorder range from GD being officially recognized as an official disorder to being seen as a pathologizing of normal behavior that may generate moral panic (2, 11, 15, 16). Another debate involves whether gaming behavior should be considered addictive, with some contending that excessive gaming may include continued engagement despite adverse consequences that may involve ineffective time management, gaming to escape from negative mood states or stress, or addictive features of games (17). Like with gambling disorder, IGD may share core components of addiction, including continued engagement despite adverse consequences, impaired control or compulsive engagement, and an appetitive urge or craving that may precede behavioral engagement (18). In the DSM-5, IGD is included under “Conditions for Further Study,” suggesting that in individuals with IGD, gaming may activate similar reward-related pathways as drugs do in individuals with drug addictions (14). Such data, along with findings related to withdrawal and significant social and cognitive impairment linked to excessive gaming, mirror those of substance-use disorders; however, differences have also been noted. Some criteria included in the DSM-5 for IGD, such as tolerance, may not be as central to IGD as to substance-use disorders. Individuals with IGD may be particularly motivated by complex and specific in-game goals, and by a fear of missing out in multiplayer games; this may be different from aspects of tolerance in substance-use disorders (19). Potential differences between IGD and substance-use disorders may be found for other criteria as more research is conducted.

With the generation of the *The International Classification of Diseases*, 11th edition (ICD-11), GD was included as a disorder due to addictive behaviors, with some researchers arguing against the inclusion (20) and others citing the relevance to personal and public health (21). Some of the debate focuses on whether there is sufficient evidence for GD to be included in ICD-11, citing the possibility of pathologizing normal behavior. However, others report that having a defined disorder should not interfere with most individuals who engage in gaming and would importantly promote generating a framework for helping those who might be experiencing harms related to gaming. Further, the inclusion of a hazardous gaming entity, like that has been used for other addictive behaviors like alcohol consumption, has been debated but may be particularly important from public health perspectives (22). These debates regarding GD share features with others in psychiatry historically (e.g., with respect to substance-use disorders) with respect to how best to define and classify disorders (23). With current categorical systems like those in the ICD-11 and DSM-5, concerns have been raised that defined entities described as discrete in reality are not distinct from others (24). This consideration may be especially concerning when behaviors exist on a spectrum from common normalcy to harmful, as is gaming.

Alternative and non-mutually exclusive dimensional approaches like the research domain criteria (RDoC) or others that focus on intermediate phenotypes may be important to consider as alternate or complementary ways of considering such behaviors or processes. Some intermediate phenotypes focus on cognitive processes or tendencies linked to brain structure and function. As such, we will now consider the neurocognitive evidence for IGD not only as it relates to substance-use disorders, but also as it relates to recreational gaming.

## Neurochemical and Functional Neural Circuitries in Internet Addiction and Gaming Disorder

Dopaminergic systems have been proposed to contribute to reward processing in IGD, and in addictions more broadly (25), although the centrality of dopamine to behavioral (26, 27) and substance (28) addictions has been questioned. Individuals with Internet addiction, compared with those without, have been reported to have lower dopamine D2-like receptor availability in the striatum and to have lower levels of striatal dopamine transporter expression (29, 30). Dopamine D2-like receptor availability in the striatum has also been inversely related to severity of Internet addiction and decreased glucose metabolism in the orbitofrontal cortex (31). All three studies included five individuals with Internet addiction so findings should be considered highly preliminary. In a possible link to genetic vulnerability, the Taq1A1 allele of *DRD2*, a gene coding for the dopamine D2 receptor, has been reported to be more prevalent in individuals with excessive/problematic gaming and associated with greater reward dependence (32) As *DRD2* is in linkage disequilibrium with *ankk1* and allelic variation in the coding region of *ankk1* has been more closely linked to addictions (e.g., alcohol-use disorders) than those in *DRD2 per se* (33, 34), questions exist as to the extent to which the observed findings may link to dopamine. Bupropion, a norepinephrine-dopamine reuptake inhibitor, may reduce cravings and cue-induced activation of the dorsolateral prefrontal cortex (DLPFC) in individuals with IGD (35). Higher scores on Internet addiction scales have been found to be associated with reduced N-acetyl aspartate in the right frontal cortex in young individuals with Internet gaming addiction (36).

Functional imaging studies have implicated cortical and striatal brain regions in IGD, particularly in males. Gaming cue-induced activity in the striatum (ventral and dorsal) has been reported to be greater in individuals with IGD as compared with those without, although activation in the left ventral striatum was negatively correlated with intensities of cue-induced cravings (37). Responses to gaming cues may change following forced immediate abstinence, and findings suggest that changes in DLPFC activation during forced immediate abstinence may in part underlie male vulnerability to IGD (38). Further, changes in functional connectivity between regions implicated in reward processing (e.g., striatum) and cognitive control (e.g., DLPFC) prior to gaming and during forced immediate abstinence may explain the progression of IGD in a gender-sensitive fashion (39). Resting-state functional connectivity between the ventral tegmental area and the nucleus accumbens, a region in the ventral striatum, has also been reported to negatively correlate with craving intensities, and with less strength in connectivity between these regions noted in individuals with IGD as compared to those without (40). The insula has been implicated in IGD with relatively decreased resting-state functional connectivity observed between regions of the insula and those like the supplementary motor areas, cingulate cortex, and superior frontal gyrus, suggesting diminished resting communication between regions implicated in interoceptive processing, craving, and other processes and the ones involved in motoric behaviors and cognitive and behavioral control (41). The processing of gaming cues and resting-state connectivity may also relate to treatments for IGD. For example, increased insula activity to gaming cues has been observed following a craving behavioral intervention in IGD, with relatively diminished connectivity between the insula (implicated in cue reactivity and interoceptive processing) and regions implicated in drug craving like the precuneus are also seen (42). Following a craving-behavioral intervention, resting-state functional connectivity was decreased between the orbitofrontal cortex and hippocampus and between the posterior cingulate and supplemental motor area (43). These findings link changes in connectivity between regions implicated in craving to those involved in memory and motoric planning processes, respectively, suggesting possible neurobiological mechanisms for a craving behavioral treatment for IGD.

Functional MRI studies may investigate neural correlates of cognitive processes including those related to control and reward/loss processing, as hypothesized to be important in IGD and other Internet-use disorders (44, 45). Individuals with IGD, as compared with those without, have demonstrated less functional connectivity within executive control regions, and this has been linked to behavioral measures of cognitive control (46). Individuals with IGD show greater frontal cortical activation during a cognitive control task than those with regular- or low-frequency game use (43). On a guessing task, an IGD group demonstrated relatively weaker frontal cortical activations during processing of losses and relatively weaker activation of cortico-striatal regions during processing of wins (47). During a risk-related decision-making task, in IGD participants there was relatively weaker modulation for experienced risk in cortical regions (DLPFC and inferior parietal areas) and increased activation of striatal and ventromedial and orbitofrontal cortices during rewarding outcomes (48). Relationships with IGD severity were noted in both studies. A separate study found that IGD subjects showed relatively decreased involvement of the inferior frontal and precentral gyri when making probabilistic choices (49). Differences in the processing of emotional cues have also been noted in IGD, with relatively blunted activation of cortico-striatal regions noted in response to negative affective cues and during emotional regulation in the striatum, insula, lateral prefrontal cortex, and anterior cingulate (50). A meta-analytic review indicated that individuals with IGD as compared with those without demonstrated relatively increased activity in the anterior and posterior cingulate cortices, caudate, and posterior inferior frontal gyrus during reward and “cold” executive functions, relatively decreased activity in the anterior inferior frontal gyrus in relation to “hot” executive functions, and relatively decreased activity in the posterior insula, somatomotor, and somatosensory cortices during reward processing (51). Together, these findings suggest neural mechanisms for disadvantageous decision-making, impaired control, and dysregulated reward processing in IGD.

The neurochemical and genetic studies of IGD highlight shared features with other addictive disorders. These shared elements suggest that IGD has similar biological underpinnings with more established addictive disorders.

## Neurocognition of Internet Gaming Disorder Compared With Other Addictions

Although relatively few studies have directly compared and contrasted neural correlates in IGD with those of substance-use disorders as has been done for gambling disorder [e.g., see Refs. (52, 54)], similarities have been noted between the neural correlates of IGD and substance-use disorders. Individuals with IGD have been reported to exhibit similarly decreased neural activity in response to losses, and increased sensitivity to cues, as in gambling and substance-use disorders (55). Responses to tobacco and gaming cues may include activations in the anterior cingulate and parahippocampus with tobacco-use disorder and IGD (56). IGD and alcohol-use disorder have been reported to share increased resting state regional homogeneity in the posterior cingulate cortex, with the IGD group showing decreased resting state regional homogeneity in the superior temporal gyrus compared with alcohol-use disorder and non-affected groups (57). While both IGD and alcohol-use disorder groups have demonstrated positive resting state functional connectivity between the DLPFC, cingulate, and cerebellum, the IGD group showed negative resting state functional connectivity between the DLPFC, temporal lobe, and striatal areas and the alcohol-use disorder groups showed positive resting-state functional connectivity between these regions (58).

The extent to which similarities may reflect common brain mechanisms across conditions may link to specific intermediate phenotypes [e.g., impulsivity, as has been implicated in brain studies across behavioral drug addictions (59)] and differences may relate to unique features of the conditions (e.g., substance effects on brain substrates) warrants additional investigation.

## Problematic Versus Regular Gaming

Recent studies have begun to include groups whose members game frequently for recreation, but do not experience negative consequences (a behavioral pattern termed “regular game use” or RGU). The use of an RGU group that reports similar amounts of time gaming as the IGD group but without the negative consequences removes a potential confound related to gaming experience that may be levied against studies of IGD and non-gaming groups. Some of the findings comparing groups with IGD and those with RGU are similar to those observed in individuals with substance-use disorders. As mentioned above, individuals with IGD as compared with those with RGU demonstrated poorer cognitive control that was associated with greater frontal activation and weaker activations of frontal and cortico-striatal regions during processing of losses and wins (47). Individuals with IGD as compared with those with RGU have been reported to exhibit less cortical thickness in the orbitofrontal cortex, inferior parietal lobule, cuneus, precentral gyrus, and right middle temporal gyrus (60). Cortico-striatal pathways also differentiate those with IGD from those with RGU with respect to craving, with IGD subjects showing greater striatal-thalamic connectivity and decreased DLPFC-superior frontal gyrus connectivity during immediate forced abstinence, with both patterns of connectivity correlating with craving intensity (39). Individuals with RGU who subsequently develop IGD have been reported to exhibit increased lentiform activation to gaming cues following gaming (61). Further, findings suggesting better white matter integrity in individuals with IGD as compared with those with RGU were reported, implicating tracts involved in processing rewards and generating sensory and motor control and linking to measures of addiction severity (62). Individuals with IGD as compared to those who are professional gamers decreased gray matter volume in cingulate gyrus and increased thalamus gray matter volume, with additional differences noted between groups, including relatively decreased volumes in the IGD and professional gaming groups relative to a non-gaming control group (63). Of note, the IGD group was more impulsive and showed more perseverative errors relative to the non-gaming group, consistent with the notion that aspects of impaired control and compulsivity may be more relevant to IGD than to other gaming and non-gaming groups (45, 64).

Beyond time spent gaming, functional impairment is an important consideration in IGD. Intermediary phenotypes, such as impulsivity and urge or craving states, are important in IGD as in other more well researched addictive disorders. These cognitive factors relate to gray and white matter measures in subjects with IGD, and more research is needed to determine whether these findings may predispose or be a consequence of problematic gaming.

## Future Directions

IGD in the DSM-5 and GD in the ICD-11 are likely heterogeneous entities, and an improved understanding of relevant individual differences will likely help diagnostic, classification, prevention, and treatment efforts. Additional direct examination of IGD as compared to other addictive disorders is warranted. Examinations targeting a broader range of neurobiological systems implicated in behavioral and drug addictions, such as glutamatergic, serotonergic, noradrenergic, GABAergic, and stress hormonal systems (65), should be conducted in IGD. Intermediate phenotypes, including impulsivity, compulsivity, positive and negative valence systems measures, social cooperation, stress responsiveness, emotional processing, and others, warrant further investigation regarding their relevance to IGD (66–69), especially as some of these features have been linked to mental health in IGD (70). Other features like escapism and gaming-specific aspects (e.g., use of avatars, discrepancies between ideal/virtual and actual self) also warrant consideration (71–73). Such research should also be extended to a broader range of Internet-use disorders (74), especially as gaming appears linked to other Internet-use behaviors like pornography viewing (75), and support for such research will be important (76). Types of gaming (including online and offline, as well as types/genres) should also be considered (77, 78), particularly as the genres of games people play the most may relate importantly to treatment outcomes (78).

Identifying individuals with IGD will be important, and the implementation of culturally sensitive and validated screening instruments will assist in this process (79). This process should be extended to additional jurisdictions and strive for briefer instruments, and such efforts are currently underway in conjunction with the World Health Organization. This will be particularly important as most people with gambling disorder do not receive treatment (80), and this is likely the case with IGD as well (81). Further research into effective treatments (especially placebo-controlled, randomized clinical trials) is needed, especially since many individuals seeking treatment for IGD continue to experience difficulties at 1- to 5-year follow-ups (82). While some data support the efficacy of specific interventions (for example, a craving behavioral intervention incorporating elements of mindfulness and cognitive behavioral therapy), randomized clinical trials are needed (42, 43). Considering the applicability of behavioral and pharmacological approaches effective in the treatments of addictions or other disorders that frequently co-occur with IGD (e.g., depression, attention-deficit hyperactivity disorder) may facilitate and accelerate this process, as has been proposed for gambling disorder in which co-occurring disorders have been reported to be helpful in selecting appropriate pharmacotherapies in the absence of medications with specific indications for gambling disorder (83). Considering potential developmental impacts of gaming and GD is also important (84). The inclusion of GD in the ICD-11 should help ensure that there is recognition of gaming-related in a subgroup of individuals in a manner that does not pathologize RGU (85), especially if functional impairment is taken into consideration (86), and the inclusion should help promote prevention, treatment, and public health efforts (21).

## Author Contributions

AV wrote the first draft in consultation with MP, and MP edited and revised the drafts. Both authors agree to the final submitted version.

## Conflict of Interest Statement

AV and MP have no conflicts of interest with respect to the contents of the manuscript. MP declares the following. M.N.P. has consulted for and advised Shire, INSYS, RiverMend Health, Addiction Policy Forum, Game Day Data, the National Council on Problem Gambling, Opiant/Lightlake Therapeutics, and Jazz Pharmaceuticals; has received unrestricted research support from Mohegan Sun Casino and grant support from the National Center for Responsible Gaming; and has consulted for and advised legal and gambling entities on issues related to addictions and impulse control disorders. He has also participated in World Health Organization meetings relating to IGD and GD. The remaining author declares that the research was conducted in the absence of any commercial or financial relationships that could be construed as a potential conflict of interest.

## Funding

MP has received support from the Connecticut State Department of Mental Health and Addiction Services, the Connecticut Mental Health Center, the Connecticut Council on Problem Gambling, and the National Center for Responsible Gaming. The funding agencies did not provide input or comment on the content of the article, and the content of the article reflects the contributions and thoughts of the authors and do not necessarily reflect the views of the funding agencies.
